# The balance between CD4+ T helper 17 and T-cell immunoglobulin and mucin domain 3 is involved in the pathogenesis and development of atrial fibrillation

**DOI:** 10.4314/ahs.v23i3.71

**Published:** 2023-09

**Authors:** Wenjing Dai, Jun Zhang, Yang Wang, Jingqun Zhou, Qiuting Dai, Jianfeng Lv

**Affiliations:** 1 Department of Cardiovasology, Affiliated Renhe Hospital of China Three Gorges University, Yichang, China; 2 Department of Critical Care Medicine, Affiliated Renhe Hospital of China Three Gorges University, Yichang, China; 3 Department of Medicine, China Three Gorges University, Yichang, China

**Keywords:** Atrial fibrillation, Th17 cells, Tim-3, Cytokines, IL-17, Gal-9

## Abstract

**Background:**

To investigate the expression of Th17, T lymphocyte immunoglobulin mucin 3 (TIM-3+) cells and their related cytokines in atrial fibrillation (AF) and their clinical significance.

**Methodology:**

A total of 90 patients with AF were divided into paroxysmal group (n=45) and chronic group (n=45), and 45 healthy volunteers were selected as the control group. The proportion of Th17 cells and Tim-3 + cells in the peripheral blood were detected. The concentrations of related cytokines in peripheral blood serum were determined. The correlation between Th17 / Tim-3+ cells and related cytokines was analysed.

**Results:**

Compared with the control group, the proportion of Th17 cells and the concentration of related cytokines (IL-17, IL-6 and Matrix metalloproteinase (MMP9)) in peripheral blood of patients with paroxysmal and chronic AF increased significantly, while the proportion of tim3 + cells and the concentration of related cytokines decreased significantly. Compared with the paroxysmal group, the proportion of Th17 cells and the concentration of related cytokines in the peripheral blood of patients in the chronic group increased significantly, while the proportion of tim3 + cells and the concentration of related cytokines decreased significantly.

**Conclusion:**

Th17 / Tim-3 + cell balance is involved in AF, and can be used as a target for AF treatment.

## Introduction

Atrial fibrillation (AF) is a common arrhythmia in clinical practice, which easily causes complications such as cardiac insufficiency, thromboembolism, cerebral apoplexy, and seriously affects the physical and mental health of patients^1^. AF occurs in approximately one-third of patients with common cardiac disorders, and increases significantly with age^2^. Therefore, the study and prevention of AF pathogenesis is one of the hotspots in clinical research. At present, most scholars believe that the occurrence and development of AF are closely related to inflammatory cells and their inflammatory factors. T helper cell 17(Th17) is a special group of CD4 + helper T cell subsets, and their main effector cytokine secreted is interleukin-17(IL-17), which plays a pro-inflammatory role in cardiovascular diseases such as viral myocarditis, rheumatic heart disease, and atherosclerosis^3^. It has been shown that Th17 cells and their related cytokines are involved in the pathological mechanism of atrial fibrillation, and the increase of Th17 cell levels and changes in inflammatory factor levels affect the immune balance of patients with atrial fibrillation^4^. Tim-3(T lymphocyte immunoglobulin mucin 3), is an inhibitory molecule expressed on the surface of T cells. And studies have shown that Tim-3 negatively regulates the function of Th17 cells, which can bind to the ligand galectin-9 (Gal-9) and then negatively regulate Th17 cell activity^5^,^6^. However, there were still a lack of research on the changes of Th17/Tim-3 + cell population and its related cytokine concentrations in the peripheral blood of patients with atrial fibrillation. Therefore, many patients with different types of AF were collected in this study to investigate the expression of Th17/Tim-3+ cells and related cytokines in patients with AF pathogenesis and provide a theoretical basis for clinical prevention and treatment of AF.

## Patients and methods

### Patients

Ninety patients who were diagnosed with AF at the Department of Cardiology, Affiliated Renhe Hospital of China Three Gorges University from January 2018 to January 2021 were selected. According to the 2010 European Society of Cardiology guidelines, the patients included in the study were divided and classified, including 45 patients with paroxysmal atrial fibrillation and 45 patients with chronic atrial fibrillation. The onset of paroxysmal AF is generally within 48 hours, up to a maximum of one week, recurrent, and self terminating; And those with chronic AF were those with AF episodes lasting greater than half a year. Forty-five healthy volunteers of the same period with normal ECG examination who were close to age were also selected as the control group. Inclusion criteria were set as follows: Patients with electrocardiogram in line with AF characteristics, that was, normal P wave disappeared, replaced by different sizes and shapes of fibrillation waves (f wave), usually V1 lead was the most obvious, the frequency of atrial fibrillation waves 350 to 600 beats/min; RR was absolutely irregular, QRS wave was generally not widened. Exclusion criteria were as follows: those with acute myocardial infarction and unstable angina pectoris, previous history of cardiothoracic surgery, valvular heart disease, congenital heart disease, thyroid dysfunction, connective tissue disease, peptic ulcer and other gastrointestinal diseases and acute and chronic infection. This study was approved by the medical ethics committee of our hospital, and all patients followed the voluntary principle.

### Main reagents and instruments

Th17 cell detection kit, monoclonal mouse anti-human CD4-Percp, Tim-3-APC antibody and lymphocyte separation solution were purchased from BD Company (Franklin Lakes, NJ, USA); (IL-17, IL-6, Tim-3, Gal-9, MMP9) enzyme-linked immunosorbent assay kit (ELISA) was purchased from Wuhan Boster Company, respectively (Wuhan, China); BD FACS Calibur model flow cytometer was purchased from BD Company (Franklin Lakes, NJ, USA); Bio-RAD-550 model microplate reader was purchased from Bole Company (Hercules, CA, USA).

## Method

### Specimen Processing

Five milliliters of peripheral venous blood were collected in EDTA anticoagulant tubes at nine o'clock in the morning in all patients. The collected blood samples were first kept at room temperature for 30 min, then centrifuged at 1500 rpm/min for 10 min, the upper serum was collected in a sterile EP tube, and frozen in a - 20 ^o^C refrigerator. . We added 1:1 volume of lymphocyte separation solution into the lower layer of blood, fully mixed well, centrifuge at 800 rpm for 20 min, and separated peripheral blood mononuclear cells according to the density gradient centrifugation method for future use.

### Detection of Th17 and Tim-3 + cells

Extracted peripheral blood mononuclear cells were resuspended and counted in RPMI 1640 complete cell culture medium. A suspension of approximately 106 cells were added to 50 µg/L of the stimulator phorbol ester and 500 µg/L ionomycin and moenomycin, and the culture were continued for 4 h in a carbon dioxide cell incubator at 5% CO2 and 37°C. At the end of the incubation, the cells were washed once with PBS phosphate buffered saline, then 5 µL of CD4-Percp and IL -17A -PE antibodies were added and incubated at room temperature for 30 min in the dark. At the end of incubation, the cell suspension were washed once with 1 mL PBS, resuspended in 150 µL PBS, and the proportion of Th17 cells (CD4+ IL-17A+) were measured by BD FACSCalibur flow cytometry (Franklin Lakes, NJ, USA).

Similarly, the counted mononuclear cells were washed once with PBS, and then incubated with 5 µL of CD4-Percp and Tim-3-APC antibodies for 30 min in the dark, followed by 1 mL of PBS for washing, and then resuspended with 150 µL PBS, and the proportion of Tim-3+ (CD4 + Tim-3+) cells in peripheral blood were detected by flow cytometry.

### Cytokine assays

50 µL of cryopreserved peripheral blood serum were collected from each patient, according to the instructions of ELISA kits for IL-17, IL-6, Tim-3, Gal-9 and MMP9. First, the standard curve were established using the standards of each cytokine in the kit, and then the concentration levels of related cytokines in the peripheral blood serum of each patient were detected by Bio-RAD microplate reader (Hercules, CA, USA).

### Statistical analysis

All data were statistically analysed using Statistical Product and Service Solutions (SPSS) 23.0 software (IBM, Armonk, NY, USA). Measurement data were expressed as mean ± standard deviation (^-^x±s), one-way analysis of variance was used for comparison among multiple groups, and SNK-q test was used for further pairwise comparison. Enumeration data were presented as n (%) and compared using x2 test. Spearman correlation analysis was used for correlation analysis. Differences were considered significant and statistically significant when P < 0.05.

## Results

### Comparison of general data of patients in each group

There were no significant differences in age, gender, basic medical history (hypertension, diabetes, hyperlipidemia), living habits (smoking, drinking), and body mass index BMI among the three groups (all P > 0.05); compared with the control group, there were significant differences in LAD and LVEF in the echocardiogram between the paroxysmal and chronic groups (all P < 0.05), while there were no significant differences in LAD and LVEF between the paroxysmal and chronic groups (all P > 0.05), as shown in Table 1.

**Table 1:** Statistics of general conditions of patients [x̅ ± s/subject (%)]

Clinical data	Control	Paroxysmal	Chronic	F/χ 2 value	P value
	group (n=45)	group (n=45)	group (n=45)		
Age (years)	61.65± 6.75	64.84± 7.79	65.25± 8.11	0.865	> 0.05
Gender (M/F)	25/20	23/22	24/21	0.973	> 0.05
History of hypertension (%)	13 (28.89)	11 (24.44)	12 (26.67)	0.811	> 0.05
History of diabetes (%)	9 (20.00)	8 (17.78)	10 (22.22)	0.854	> 0.05
History of hyperlipidemia (%)	12 (26.67)	13 (28.89)	15 (33.33)	0.525	> 0.05
Alcohol consumption (%)	5 (11.11)	7 (15.56)	6 (13.33)	0.690	> 0.05
Smoking (%)	8 (17.78)	6 (13.33)	9 (20.00)	0.621	> 0.05
BMI (kg/m 2)	26.75± 3.36	25.94± 3.47	26.11± 3.85	0.894	> 0.05
LAD (mm)	34.14± 3.52	38.45± 3.95	39.84± 4.11	0.024	< 0.05
LVEF (%)	71.35± 4.88	67.58± 5.13	67.27± 5.95	0.399	< 0.05

### Changes in the proportion of Th17 cells in the peripheral blood of patients in each group

**Figure 1:**
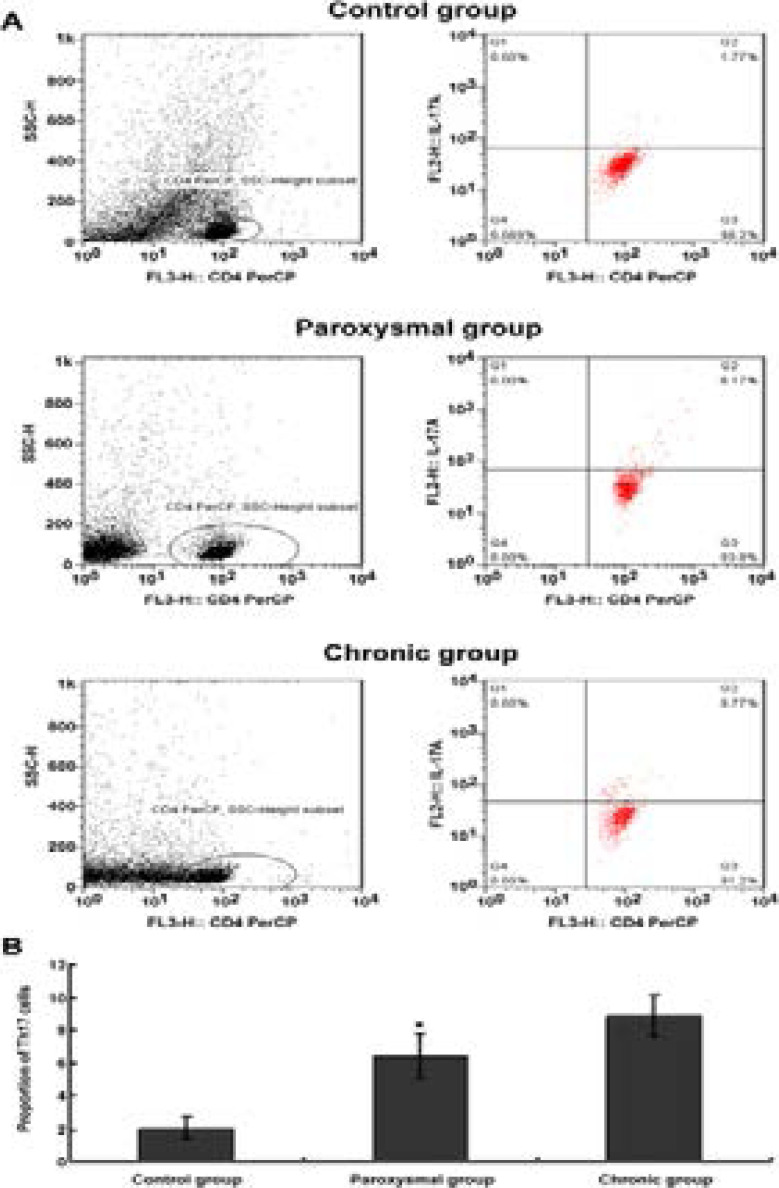
Changes in the proportion of Th17 cells in peripheral blood of patients in each group. * Indicates P < 0.05 compared with control; # indicates P < 0.05 compared with paroxysmal group. (A) Typical diagram of flow cytometric detection of Th17 cells in CD4 + IL-17A + of patients in each group; (B) Statistics of proportion of Th17 cells in patients in each group

Flow cytometry was used to detect changes in the proportion of Th17 cells (CD4 + IL -17A +%) in the peripheral blood of patients in each group, and the results are shown in Figure 1. After statistics, compared with the control group, the proportion of Th17ells in the peripheral blood of patients with atrial fibrillation in the paroxysmal and chronic groups increased significantly [(2.05± 0.67)% vs (6.45± 1.32)% vs (8.89± 1.28)%], and the difference was statistically significant (F = 15.211, P < 0.05); while further pairwise comparison showed that the proportion of Th17 cells in the peripheral blood of patients with atrial fibrillation in the chronic group was also significantly higher than that in the paroxysmal group, (t = 11.248, P < 0.05).

### Changes in the proportion of Tim3 + cells in the peripheral blood of patients in each group

**Figure 2:**
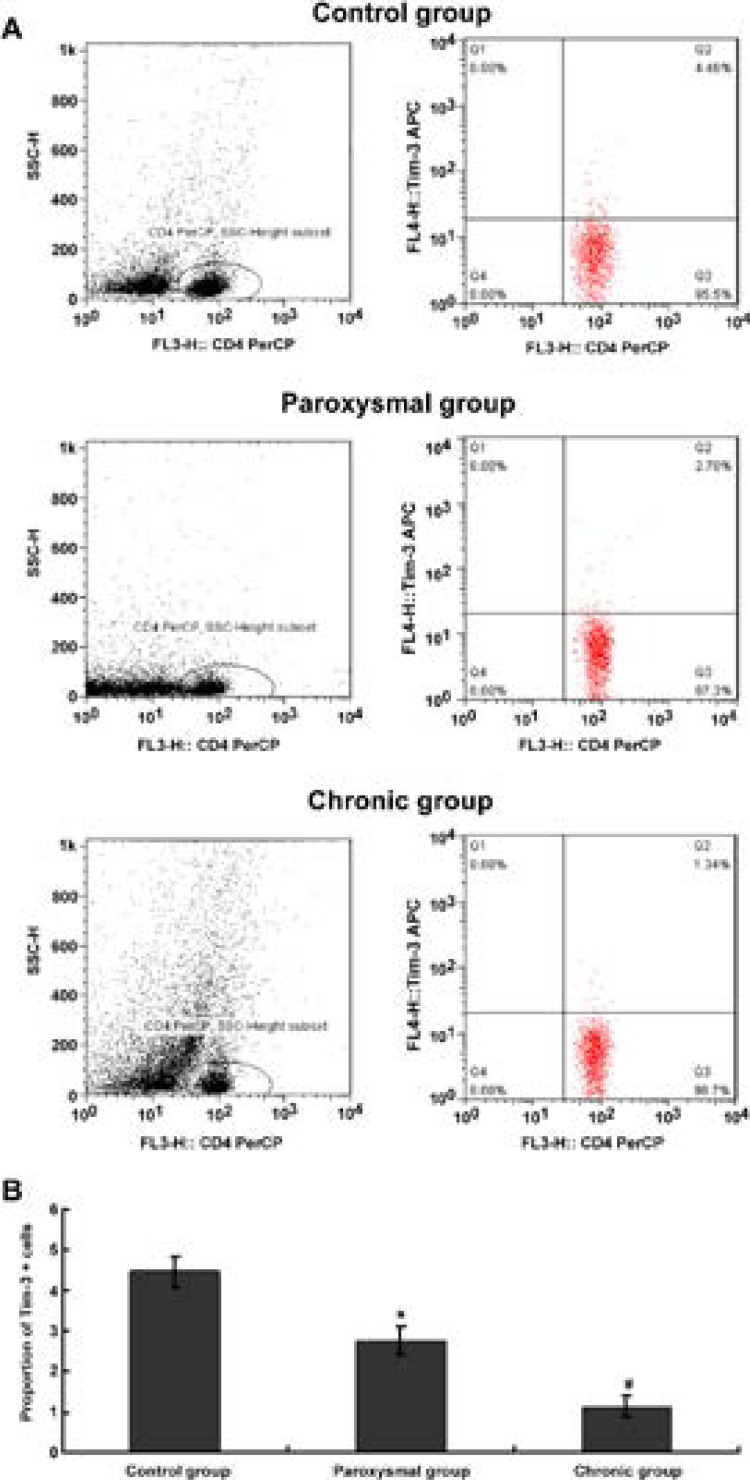
Changes in the proportion of Tim-3 + cells in peripheral blood of patients in each group. * Indicates P < 0.05 compared with control; # indicates P < 0.05 compared with paroxysmal group (A) Typical diagram of flow cytometric detection of Tim-3 + cells of CD4 + Tim-3 + in each group; (B) Statistics of the proportion of Tim-3 + cells in each group

Flow cytometry was used to detect changes in the proportion of Tim-3+ cells (CD4+ Tim-3+%) in the peripheral blood of patients in each group, and the results are shown in Figure 2. After statistics, compared with the control group, the proportion of Tim-3+ cells in the peripheral blood of patients with atrial fibrillation in the paroxysmal and chronic groups were significantly decreased [(4.45± 0.38)% vs (2.75± 0.34)%, vs (1.12± 0.26)%], and the difference was statistically significant (F= 13.287, P < 0.05); while further pairwise comparison showed that the proportion of Tm-3 cells in the peripheral blood of patients with atrial fibrillation in the chronic group was also significantly lower than that in the paroxysmal group, (t = 15.627, P < 0.05).

### Changes of Th17 / Tim-3 + cell ratio in peripheral blood of patients in each group

**Figure 3:**
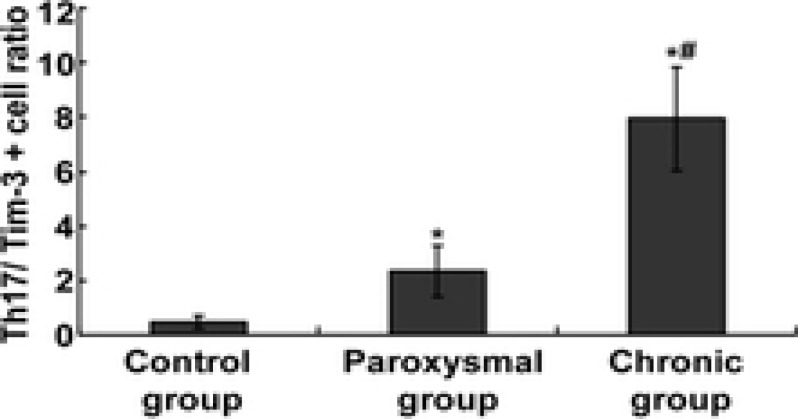
The changes the Th17 / Tim-3 + cells ratio in each group. * Indicates P < 0.05 compared with control; # indicates P < 0.05 compared with paroxysmal group

Compared with the control group, the ratio of Th17 / Tim-3+ cells in the peripheral blood of patients in paroxysmal group and chronic group were significantly higher [(0.46 ± 0.23) vs (2.35 ± 0.95) vs (7.94 ± 1.92)], and the difference was statistically significant (F = 25.333, P < 0.05); Furthermore, the ratio of Th17 / Tim-3+ cells in peripheral blood of patients with AF in chronic group was significantly higher than that in paroxysmal group (t = 28.394, P < 0.05). And the results are shown in Figure 3.

### Changes in serum cytokine (IL-17, IL-6, Tim-3, Gal-9, MMP9) concentrations in each group of patients

The results of ELISA for changes in the concentrations of related cytokines (IL-17, IL-6, Tim-3, Gal-9, MMP9) in the peripheral blood sera of patients in the three groups are shown in Table 2. According to statistics, compared with the control group, the concentrations of IL-17, IL-6 and MMP9 cytokines in the peripheral blood serum of patients with atrial fibrillation in the paroxysmal and chronic groups were significantly increased (F = 16.120, 27.476 and 19.934, respectively, all P < 0.05), while the concentrations of Tim-3 and Gal-9 cytokines were significantly decreased (F = 15.395 and 18.141, respectively, all P < 0.05); further comparison showed that the concentrations of IL-17, IL-6 and MMP9 in the peripheral blood serum of patients with atrial fibrillation in the chronic group were also significantly higher than those in the paroxysmal group (t = 11.024, 9.005 and 10.335, all P < 0.05), while the concentrations of Tim-3 and Gal-9 cytokines were also significantly lower than those in the paroxysmal group (t = 7.255 and 6.146, all P < 0.05).

**Table 2:** Concentration and expression of related cytokines in peripheral blood serum of patients in each group (n=45, x̅±s)

	IL-17 (pg/mL)	IL-6 (pg/mL)	Tim-3 (pg/mL)	Gal-9 (pg/mL)	MMP9 (pg/mL)
Control group	85.46 ± 8.94	98.95 ± 3.11	51.24 ± 8.13	22.35 ± 5.45	135.45 ± 18.14
Paroxysmal group	138.23 ± 13.24 *	224.44 ± 18.92 *	38.68 ± 6.67 *	17.24 ± 4.04 *	241.34 ± 20.09 *
Chronic group	194.25 ± 15.37#	264.58 ± 21.24#	16.97 ± 4.35 #	13.33 ± 3.37#	298.47 ± 28.34#

* Indicates *P* < 0.05 compared with control

# indicates *P* < 0.05 compared with paroxysmal group

### Correlation analysis of th17/tim-3 + cells and cytokines (il-17, il-6, tim-3, gal-9, mmp9) in peripheral blood of AF patients

After statistical analysis, the proportion of Th17 cells and Tim-3+ cells in the peripheral blood of AF patients was negatively correlated (r = 0.674, P < 0.05); the proportion of Th17 cells in the peripheral blood of AF patients was positively correlated with IL-17, IL-6, Tim-3, Gal-9, and MMP9 cytokine levels (r = 0.774, 0.468, 0.456, all P < 0.05), and negatively correlated with IL-17, IL-6, Tim-3, Gal-9, and MMP9 cytokine levels (r = − 0.511, − 0.498, all P < 0.05); the proportion of Tim-3 + cells was also positively correlated with Tim-3 and Gal-9 cytokine levels (r = 0.811, 0.512, all P < 0.05); the proportion of Tim-3 + cells was negatively correlated with IL-17, IL-6, and MMP9 cytokine levels (r = − 0.434, − 0.493, − 0.507, all P < 0.05).

### Correlations between associated cytokines and echocardiographic parameters in peripheral blood of patients with AF

After statistical analysis, Th17 cells and their related cytokines (IL-17, IL-6, MMP9) in the peripheral blood of AF patients were positively correlated with LAD (r = 0.493, 0.515, 0.385, 0.406, all P < 0.05), while Tim-3 + cell ratio and its related cytokines (Tim-3, Gal-9) were negatively correlated with LAD (r = − 0.484, − 0.511, − 0.381, all P < 0.05). Th17 cells and their related cytokines (IL-17, IL-6, MMP9) in the peripheral blood of AF patients were negatively correlated with LVEF (r = − 0.462, − 0.457, − 0.313, − 0.375, all P < 0.05), while Tim-3 + cell ratio and their related cytokines (Tim-3, Gal-9) were positively correlated with LVEF (r = 0.428, 0.416, 3.724, all P < 0.05).

## Discussion

The prevention and treatment of AF in atrial fibrillation is the focus of clinical cardiovascular disease research. Current studies suggest that in addition to age, hypertension, coronary heart disease, valvular disease and other causes, the causes of AF are also associated with other risk factors such as increased inflammatory cells, inflammatory factor burst, metabolic disorders7. In recent years, it has been found that abnormal infiltration of a variety of immune cells, such as T lymphocytes, can be detected in the atrial tissue of AF patients, and the immune subtraction of these infiltrates is involved in atrial fibrosis, which in turn induces the production of atrial fibrillation^8^. Th17 cells are a subset of envoy CD4 + cells, and it has also been found that Th17 cells and their secreted cytokine IL-17 are involved in the fibrotic process in a variety of organs^9^.

In rat models of AF, a significant increase in the proportion of Th17 cells has been widely observed and suggests that Th17 cells are involved in the progression of AF pathology^10^. Increased tendency of Th17/Treg immune cell balance toward Th17 cell differentiation has also been observed in patients with atrial fibrillation, which leads to enhanced inflammatory response^11^. In this study, flow cytometry was used to directly detect the changes of Th17 cell ratio in peripheral blood of patients with paroxysmal and chronic AF. The study also found that the proportion of Th17 cells in peripheral blood of patients with AF was significantly increased, while the Th17 level in patients with chronic AF was significantly higher than that in patients with paroxysmal AF, indicating that Th17 cells may promote the occurrence of atrial fibrillation. Current studies have found that Tim-3 negatively regulates T cell function and is involved in the immune regulation of Th, Tim-3 binds to Gal-9 and negatively regulates Th17 cell functional status, and Tim-3 is involved in the protection of tissue injury by regulating Th17/Treg imbalance^12^,^13^. Therefore, it is necessary to investigate the expression pattern of Tim-3 in AF patients to reveal its relationship with AF development. Tim-3 is expressed on the surface of helper T cells (CD4 +) and therefore marks the Tim-3 + cell population in T lymphocytes by CD4 + Tim-3 +^14^,^15^. In this study, the proportion of Tim-3 + cells in peripheral blood of patients with paroxysmal and chronic AF was significantly decreased, and the level of Tim-3 + cells in peripheral blood of patients with chronic AF was lower than that of patients with paroxysmal AF, indicating that Tim-3 + cell population is also involved in the pathological progression of AF and is closely related to AF, which may be a therapeutic target for the improvement of AF.

A large number of studies have confirmed that inflammatory factors play an important role in the development of AF, such as IL-17, IL-6, IL-22 are significantly increased in patients with chronic atrial fibrillation, and have been suggested that inflammatory factors are involved in the process of AF^16^. IL-17 is an effector cytokine of Th17 cells, and its significant role is involved in the induction and mediation of inflammatory responses. IL-17 is involved in the pathological process of a variety of cardiovascular diseases, and is also closely related to the development of AF^17^. There is also evidence that IL-6 is also one of the factors secreted by Th17 cells, and Th17 can cause inflammatory infiltration of the atria leading to AF by secreting IL-6^18^. The natural ligand of Tim-3 is Gal-9, and it has been reported that Tim-3 and Gal-9 interact to inhibit Th1 and Th17 responses after ligand binding and induce peripheral immune tolerance in the body^19^. Matrix metalloproteinase MMP9 plays a key role in myocardial fibrosis and has also been shown to induce a significantly higher inflammatory response in atrial issues of AF patients^10^. In this study, we detected the concentration changes of Th17/Tim-3 + cell-related cytokines (IL-17, IL-6, Tim-3, Gal-9, MMP9) in the peripheral blood of AF patients using ELISA and found that IL-17, IL-6, and MMP-9 cytokine levels were significantly increased in the serum of AF patients, while Tim-3 and Gal-9 levels were significantly decreased, consistent with the above findings, and also confirmed that Th17/Tim-3 + -related cytokines (IL-17, IL-6, Tim-3, Gal-9, MMP9) are involved in the pathological progression of AF.

At the same time, this study compared the correlation between the proportion of Th17 cells and Tim-3 + cells in the peripheral blood of AF patients and found a significant negative correlation between Th17 cells and Tim-3 + cells, reconfirming that Tim-3 can negatively regulate Th17 cells in AF patients. In this study, the proportion of Th17 cells was positively correlated with IL-17, IL-6, and MMP9, indicating that the increase of Th17 cells in the peripheral blood of AF patients can induce the production of inflammatory cytokines IL-17, IL-6, and MMP9, which in turn affects AF, and also indicating that Th17 cells can be used as one of the risk factors for AF. However, the proportion of Tim-3 + cells in peripheral blood of AF was positively correlated with the levels of Tim-3 and Gal-9 factors, which also suggested the role of negative regulation of Th17 cells after the binding of Tim-3 and ligand gal-9. However, Th17 cells and their related cytokines (IL-17, IL-6, MMP9) were positively correlated with LAD and negatively correlated with LVEF in AF patients, while Tim-3 + cells and their related cytokines (Tim-3, Gal-9) were negatively correlated, again indicating that Th17/Tim-3 + cells are involved in the pathological progression of AF and can be a key target for the treatment of atrial fibrillation. However, based on the present study only collected a total of 90 clinical AF patients, there is still insufficient statistical analysis of the data, so it is necessary for our next study to expand the collection of AF cases and to perform statistical analysis of the data to make Th17 / Tim-3 + cells and pathological relevance of AF more reliable for clinical application.

## Conclusion

In summary, this study revealed that Th17 cells and their related cytokines (IL-17, IL-6, MMP9) were significantly increased, while Tim-3 + cells and their related cytokines (Tim-3, Gal-9) were significantly decreased in the peripheral blood of AF patients, and Th17 cells and their cytokines were negatively correlated with Tim-3 + cells and their cytokines, and involved in the development of the course of AF. Clinical regulation of Th17/Tim-3 cell balance may facilitate the diagnosis and treatment of AF and inhibit its development.

## Data Availability

The datasets used and analysed during the current study are available from the corresponding author on reasonable request.

## References

[1] Kirchhof P, Calkins H (2017). Catheter ablation in patients with persistent atrial fibrillation. Eur Heart J.

[2] Denham NC, Pearman CM, Caldwell JL, Madders G, Eisner DA, Trafford AW (2018). Calcium in the Pathophysiology of Atrial Fibrillation and Heart Failure. Front Physiol.

[3] Wang X, Fan H, Wang Y, Yin X, Liu G, Gao C (2021). Elevated Peripheral T Helper Cells Are Associated with Atrial Fibrillation in Patients with Rheumatoid Arthritis. Front Immunol.

[4] Wang L, Chen W, Kang FB, Zhang YH, Qi LL, Zhang YZ (2021). Blood transfusion practices affect CD4(+) CD25(+) FOXP3(+) regulatory T cells/T helper-17 cells and the clinical outcome of geriatric patients with hip fracture. Aging (Albany NY).

[5] Chen Y, Chang G, Chen X, Li Y, Li H, Cheng D (2020). IL-6-miR-210 Suppresses Regulatory T Cell Function and Promotes Atrial Fibrosis by Targeting Foxp3. Mol Cells.

[6] Li N, Brundel B (2020). Inflammasomes and Proteostasis Novel Molecular Mechanisms Associated with Atrial Fibrillation. Circ Res.

[7] Oesterle A, Liao JK (2019). The Pleiotropic Effects of Statins - From Coronary Artery Disease and Stroke to Atrial Fibrillation and Ventricular Tachyarrhythmia. Curr Vasc Pharmacol.

[8] Hoffmann J, Mas-Peiro S, Berkowitsch A, Boeckling F, Rasper T, Pieszko K (2020). Inflammatory signatures are associated with increased mortality after transfemoral transcatheter aortic valve implantation. Esc Heart Fail.

[9] Rahmati Z, Amirzargar AA, Saadati S, Rahmani F, Mahmoudi MJ, Rahnemoon Z (2018). Association of levels of interleukin 17 and T-helper 17 count with symptom severity and etiology of chronic heart failure: a case-control study. Croat Med J.

[10] Fu XX, Zhao N, Dong Q, Du LL, Chen XJ, Wu QF (2015). Interleukin-17A contributes to the development of post-operative atrial fibrillation by regulating inflammation and fibrosis in rats with sterile pericarditis. Int J Mol Med.

[11] Yue H, Gu J, Zhao X, Liang W, Wu Z (2021). Role of the interleukin-17 pathway in the pathogenesis of atrial fibrillation associated with inflammation. Arch Med Sci.

[12] Zhao Y, Li X, Yu D, Hu Y, Jin W, Qin Y (2020). Galectin-9 is required for endometrial regenerative cells to induce long-term cardiac allograft survival in mice. Stem Cell Res Ther.

[13] Wu H, Tang S, Zhou M, Xue J, Yu Z, Zhu J (2021). Tim-3 suppresses autoimmune hepatitis via the p38/MKP-1 pathway in Th17 cells. Febs Open Bio.

[14] Hu X, Zhu Q, Wang Y, Wang L, Li Z, Mor G (2020). Newly characterized decidual Tim-3+ Treg cells are abundant during early pregnancy and driven by IL-27 coordinately with Gal-9 from trophoblasts. Hum Reprod.

[15] Racca V, Torri A, Grati P, Panzarino C, Marventano I, Saresella M (2020). Inflammatory Cytokines During Cardiac Rehabilitation After Heart Surgery and Their Association to Postoperative Atrial Fibrillation. Sci Rep-Uk.

[16] Seo C, Michie C, Hibbert B, Davis DR (2020). Systematic review of pre-clinical therapies for post-operative atrial fibrillation. Plos One.

[17] Qiu H, Ji C, Liu W, Wu Y, Lu Z, Lin Q (2018). Chronic Kidney Disease Increases Atrial Fibrillation Inducibility: Involvement of Inflammation, Atrial Fibrosis, and Connexins. Front Physiol.

[18] Wu N, Xu B, Liu Y, Chen X, Tang H, Wu L (2016). Elevated plasma levels of Th17-related cytokines are associated with increased risk of atrial fibrillation. Sci Rep-Uk.

[19] Li J, Zuo K, Zhang J, Hu C, Wang P, Jiao J (2020). Shifts in gut microbiome and metabolome are associated with risk of recurrent atrial fibrillation. J Cell Mol Med.

